# Lessons to be learned from the oldest community psychiatric service in the world: Geel in Belgium

**DOI:** 10.1192/pb.bp.115.051631

**Published:** 2016-08

**Authors:** Henck P. J. G. van Bilsen

**Affiliations:** 1 Waikato District Health Board, New Zealand

## Abstract

This article reviews the family foster care model practised in the small Belgian town of Geel. A historical introduction is followed by a description of a family foster care project in its current form. Issues are raised as to whether the current culture of care pathways, managed care, payment by results and an emphasis on ‘cure’ are conducive to recovery as promoted by the recovery model. Finally, the lessons from Geel are summarised and it is argued that there is much that can be learned from this way of working to support the recovery movement.

Recovery and rehabilitation have become fashionable words in mental health and psychiatry. In the current Zeitgeist this emphasis on recovery is accompanied with an ever increasing emphasis on further professionalisation and bureaucratisation of mental healthcare.

The achievements in mental healthcare in the past 50 years are enormous. We have better drugs and better psychosocial treatments are available for our patients. However, with the vast improvements came two trends that the family foster care project in Geel (Belgium) has not succumbed to.

The first problem is over-bureaucratisation of healthcare. Problems with the quality of care are countered with greater emphasis on demonstrating (with paper or digital records) that the required care is provided. Activity focused on bringing people back to health is in constant competition with completing the paperwork that is seen as evidence that carers are doing the right thing. Thus, tension exists between providing good-quality care, care that works and is evidence based, and care that constantly demonstrates on paper or by digital means that the rules are followed. Unfortunately, all too often the perspective that ‘the care given’ only counts when it is written down prevails.^1^

In this article I will attempt to make a case for a style of recovery ‘par excellence’. Not recovery that demands but recovery that accepts what is and invites the patient to find their own and perhaps very slow path to better health. Geel in Belgium, the oldest therapeutic community in Europe, may be such an example of real, patient-focused recovery. The Geel model does not rely on large amounts of rules and regulations and staff do not need to administratively demonstrate that these rules are followed. In the Geel model, people, patients and carers are allowed to ‘be’ and discover their mutual road to understanding and recovery.

The second problem that emerged with advances in psychiatric treatments is the presumption that all patients will get better following predetermined pathways and time frames. The current trend in mental healthcare seems to be focused around care that follows predetermined pathways, with a recommended length of stay and session-limited outpatient psychological therapy. Unfortunately, there will always be people who do not fit these care pathways and protocols owing to the severity and chronicity of their psychiatric problems. These patients do not fit the profile of ever-improving patients who can be rehabilitated to a level of independent functioning within set time frames. These patients seem to be ‘broken’ people who in an atmosphere of firm kindness and with opportunities to ‘be as they are without a demand for change’ might recover far more than in a system that demands of them to participate in psychosocial treatment. What we are offering many patients with a chronic mental illness is a pressure cooker model of recovery. The Geel model seems to be respectfully offering a slow cooker model.

## Recovery

Psychosocial rehabilitation and recovery have as their mission the acknowledgement of the patient as a person and enabling them to become a contributing citizen again. Recovery interventions are focused on teaching people how to manage their mental illness and ensuing limitations. The aim is to achieve an optimal integration in society.

The emergence of a recovery model in the 1990s in the USA has been considered to have been the result of the physical disability movement and deinstitutionalisation within psychiatry.^2^ New Zealand has long followed this philosophy in its approach to mental healthcare. In the UK, the concept of recovery has been built on the legislative foundations of disability rights, anti-discrimination and the civil rights movements of the 1960s and 1970s. The concept of recovery emerged from those people who had first-hand experience of mental health difficulties^3^ and is arguably a political response to an unsatisfactory mental health system that focuses on maintenance of disorders.^4^ In the mental health system we do things to and with patients in order to offer treatment, but frequently these ‘treatments’ cause more problems: the acute stress of being admitted to hospital far away from home; the mind-disabling effects of electroconvulsive shock therapy; medications with side-effects for which new medications need to be prescribed; psychological therapies that have a re-traumatising impact on patients. This is eloquently highlighted in Breggin's work, most notably in *Brain-Disabling Treatments in Psychiatry*.^5^

Today ‘recovery’ is a term that is synonymous with mental healthcare. There is no one single definition of recovery as it is deemed to be a personal and individual concept. However, there are a number of definitions which have sought to capture the philosophy of recovery. Recovery is,

‘a deeply personal, unique process of changing one's attitudes, values, feelings, goals, skills, and/or roles. It is a way of living a satisfying, hopeful, and contributing life even with limitations caused by illness. Recovery involves the development of new meaning and purpose in one's life as one grows beyond the catastrophic effects of mental illness.’^6^

Deegan^7^ says of recovery that ‘The goal of recovery is not to become normal. The goal is to embrace the human vocation of becoming more deeply, more fully human.’

The extent to which we are achieving a true recovery-focused model in UK mental health services is debatable. However, one place where this is not in doubt is the family foster care project in Geel.

## Geel: the oldest therapeutic community in Europe

Van Dale, the main Dutch language dictionary,^8^ lists under the word ‘Geel’: ‘Expression: bound to go to Geel, to come from Geel: to be mentally ill’. A more humorous expression about the people of Geel is: ‘Half Geel is entirely crazy: and entire Geel is half crazy’.^9^

The small provincial town of Geel in Flemish Belgium is host to a centuries-old mental health project. Belgium is generally famous for beers, fries, chocolate and the interesting form of federalised government it adopted. Geel is a city with around 35 000 inhabitants located in the Belgian province of Antwerp. On a visit to Geel and the family foster care system in 1977 with a small group of fellow psychology students, I found many things wrong with the Geel system. Perhaps due to the arrogance or naivety of youth, I thought it was pure ‘neglect’ that most of the patients cared for were not receiving some form of intensive psychological therapy. Another sign of ‘neglect’ was that many patients were just doing what they wanted to do and were not involved in what we saw as meaningful occupational or psychological therapy. To our horror, we also learned that the families that were looking after these patients hardly received any form of training at all. As a group, we imposed our own youthful standards on the Geel project and decided that it was not good enough! With the hindsight of experience (and age) I can now see how wrong we were. Geel truly is the first psychiatric therapeutic community in Europe.^10,11^

In this article I would like to introduce the Geel family foster care system to a wider audience and focus on what we can learn from Geel.

### Origins of family foster care in Geel

Since the year 600, so the story goes, and certainly since the 13th century, Geel has been a haven for those with mental illness.^12^ The first written patient records from Geel are dated 1693. The selection of Geel as a locality for the treatment of people with mental illness starts with the story of St Dymphna and her acceptance by the church as the patron saint of the mentally ill. Dymphna was an Irish princess, raised in the Christian faith, from what is now known as County Tyrone. A conflict erupted between the princess and her father. She fled her homeland but was finally caught and murdered by her father, in a fit of madness, in the forest near the present site of Geel. The day was 30 May 600 ad, and Dymphna was buried at the site, where the church of St Dymphna was later erected. This was at a time when the symptoms of what we now call mental illness were attributed to being possessed by the devil. This was combined with the belief that this ‘possession’ could be lifted if the person was brought in contact with religious relics of certain saints. St Dymphna was supposed to be such a saint.^13^

The fact that Dymphna had resisted the clearly ‘possessed’ king made her attractive as a saint who could cure possession. During pilgrimages to Dymphna's tomb it was hoped she might intercede on behalf of the ‘possessed’ souls and end their suffering. Miraculous cures were reported to occur in Geel. With the dissemination of Dymphna's legend, Geel became increasingly renowned as a centre for the cure of the ‘possessed’ (mentally ill). Inhabitants of Geel and farmers from the surrounding villages started to offer accommodation to the pilgrims/patients during their stay in Geel. Some pilgrims stayed on after they had completed their religious rituals and so the family foster care tradition was born. Before the first psychiatric hospital was established in the early 15th century in Valencia in Spain, the family foster care system was already in place in Geel.^14^ The Geel initiative was not based on the initiative of a churchman as in Valencia or a physician as in Pinel's hospital in Paris, but was an initiative from the people (peasants and burghers of Geel).

The church continued guardianship of this project until 1852 when the work was taken over by the state and placed under medical direction. The high point of the Geel project came just before the Second World War, when almost 4000 patients were looked after in the family foster care system. Most patients were Belgian, but many were Dutch, French, and even English. There were also several Spaniards and Russians. Patients represented the complete range of mental health problems. The cost of family foster care was paid for by the patient's relatives or by the community from which the patient came. There was some kind of selection with respect to families that could look after these patients. To be qualified (or ‘certified’ in Geel-ian terms) required that the family had a good record, meaning that no family member had ever been in legal or moral trouble (behaving in a manner that went against the moral code of the time). Certification was a matter of pride and social standing; not to be certified meant that something was not right with the family. The desire to do this work was instilled long ago, and it became a *raison d'être*, a tradition handed down for generations. Certain families were known for the adept guidance of certain types of patients, a point of considerable importance and source of personal pride.

An infirmary where patients could be admitted if they became completely unmanageable or ill was also set up. The patients were returned to their boarding homes after recovery. If boarding-out was inadvisable, patients were transferred to a closed mental hospital.

On admission to the infirmary the normal course was a brief period of observation. Afterwards, the medical staff and representatives from the town of Geel decided the best course for the individual. It was all quite informal; one of the staff might opine that the patient should best go with a certain family. If there was consensus, the transfer was made. Occasionally, but not often, several shifts from homes were necessary early in the colonial life of an individual until the patient found their proper niche. There were more homes available than there were patients. Geel was able to retain 80% of all persons sent there for boarding care.

## Geel now

The idea that mental health crises might be managed in a setting other than a traditional in-patient ward has a long history. However, we know remarkably little about such alternatives, their availability and distribution, the service models they employ, the clinical populations they serve, and the outcomes they achieve. A key question is whether, within a modern mental health system, alternatives to traditional in-patient care can achieve good outcomes for patients whose clinical and social difficulties are so severe that in-patient admission would otherwise be required. Is Geel such a place?^15–18^

Geel in the 21st century has developed into a professional and progressive institute offering mental health treatment. On its website it states: The ‘Openbaar Psychiatrisch Zorgcentrum (OPZ) – Geel’ is an integrated psychiatric centre which coordinates three autonomous divisions. One of these divisions is the family foster care project. The OPZ helps every human being in need of psychiatric care, regardless of his/her gender, background and beliefs (www.opzgeel.be/nl/home/htm/intro.asp).

### Profile of the patients

Currently, the number of patients looked after by the family foster care project is just above 500. The patients suitable for family care typically have chronic psychiatric problems such as schizophrenia or psychotic, personality and mood disorders. There are three target groups: young people, adults and older people. At the moment there are 200 foster care families.^19^

### Criteria for acceptance

It is important that the acute phase of a psychosis or other problems has passed. Aggressive behaviour needs to be reasonably well under control and there is no history of sexual offences or serious crime. A significant group of patients have comorbid intellectual disabilities. Patients should be capable of emotional attachment, some form of communication and doing things independently.^20^

### Admission procedure

The admission procedure seems to be one of finding the right match between patient and foster family. This is not a one-sided process, rather a three-way system: the patient, the foster family and the clinicians all need to have confidence in the ‘match’.

When patients first come to Geel, they are formally admitted to the hospital. During the day they attend a programme in the Observation House where a maximum of eight patients live together with two coaches. Patients have domestic duties (shopping, cooking, ironing, gardening), work on their general skills and find out what it means to live together, assume responsibilities and share. Meanwhile, coaches find out what the patients' wishes, desires and expectations are and they monitor and assess their behaviour and potential. The coaches regularly report to the admission team headed by the medical director/psychiatrist.

From the point of view of patients, who have possibly spent years living in a hospital ward, the potential advantages of living in a family and participating in a larger community are endless.

### Beyond admission

From the moment the patient starts to be part of a family, they regain an identity. They are a member of a family again, possibly with children, grandchildren, aunts and uncles. They belong to a social network (neighbours, friends, acquaintances, etc.). They have responsibilities (doing the dishes, laying the table, peeling the potatoes, unpacking the shopping, mowing the lawn, etc.).

### Not cure but care

There are expectations, but no demands. Patients are allowed to function on their optimal level. If someone is capable of ‘pottering around’ in the garden of the foster family then that is OK; just as it is OK for another patient to join the local football club. Accepting what is, not demanding what should be, seems to be the motto.

Often people live in the same family for years. The average stay in the family care programme is currently 30 years. Thanks to their general well-being and sense of security, levels of medication often drop significantly.

Another crucial factor is the support of the community of Geel. People are used to having psychiatric patients around and are eager to help. Local schools, the arts centre and sports associations are keen to get involved.

### Profile of the families

Foster families are from a wide range of backgrounds. What is important is that the homes are caring, stable and able to cope with stressful situations. Apart from that a regular income is required and sufficient space to lodge one or more guests. Of course, the children need to agree and everyone needs to be fully aware of what it means to live with a psychiatric patient.

Families do not receive training about psychiatric illnesses. The secret of the success of family care lies in the ordinary, common-sense approach. The aim seems to be to surround the patient with normal expectations, normal demands in an atmosphere I would like to describe as radical compassion and kindness, beautifully demonstrated in the DVD that accompanies the Goosens & van de Walle book.^20^

In recognition of their commitment, families receive a monthly allowance of approximately €500, which is of course significantly less than conventional hospital admission rates.

In return for their care, foster parents receive company, friendship and a helping hand from their guests. In some cases roles are eventually reversed: patients attend to their ageing foster parents or look after them in difficult times (e.g. death or illness of a partner).^21^

### Support team

Twelve district nurses are central to psychiatric family foster care. They each look after the best interests of approximately 40 patients. They act as contacts, confidents and advocates for the patient and family. They monitor the interaction between the patient and the family. They regularly visit the family to ensure that everything is going smoothly and to deliver the prescribed medication. In case of problems, they mediate, report on the patient's behaviour or intervene if necessary. In difficult times there is daily contact. Somebody is on ‘stand-by’ to help around the clock.

### Work, occupation and leisure

Half of all patients in the family care system work at the OPZ or attend occupational therapy. The other half remains at home. The type of work patients do depends on their age, general skills and personal inclination. The OPZ provides jobs in sheltered workshops (semi-industrial work, bike shop, bookbinding, printing shop, woodcarving, gardening) and in local businesses (shop, gardening centre) for those patients who can handle the responsibility and are eager to work.

Apart from that, the OPZ runs three community centres in the city which carefully balance work and leisure. For the older and infirm patients, the OPZ has set up a club which aims to create a pleasant ambience and entertainment. A team of people are in charge of leisure activities, day trips and travel abroad, whereas another team of dedicated sportsmen and -women organise weekly sports activities tailor-made for every group: ‘Our patients are keen tennis players, challenge local soccer teams; take part in fundraising drives like the swimming marathon, etc. It is amazing how fit some of them are at the blessed age of seventy’.^19^

### Length of stay

Family foster care is in general not a short-term option. Verbiest *et al*^19^ provide an overview that shows that in 2014 the majority of the patients had been in family foster care for more than 20 years, with two patients of more than 75 years. This is of course only possible from a multi-generational perspective: children and grandchildren of the original foster parents continue to offer a home to their *kostganger* (boarder).

### The cost

The total cost of family foster care is at the moment €47 per day. For comparison, the day price in a psychiatric hospital in Belgium is €280 and supported living is €90 (2014 figures).^19^ Family foster care certainly does not break the bank. It is a more than affordable provision of mental healthcare, with a heart and in the heart of a community.^17^

## The ‘secret’ to Geel's success

Following a 10-year study of Geel's foster family care system, the Geel Research Project sociologist Leo Srole observed that the foster family takes in a stranger who becomes a functioning member within the family structure. The role of the family as caretaker, teacher, natural supportive parent and behavioural model allows the boarder to function in the ‘normal’ social world in spite of their illness. Geel psychologist Marc Godemont,^22^ after 28 years in Geel's mental healthcare system, describes what he believes to be the ‘secret’ of Geel's success, i.e. factors that are present today and, to some degree, have probably always been present:

- Geel acknowledges the idiosyncratic needs of boarders
- the community responds to those needs by providing social opportunities and meaningful work in the community
- people with mental illness in Geel are members of both a foster family and a foster community.

From a theoretical perspective, the community of Geel has created a learning environment whereby people with mental illness are exposed to as much ‘normal’ behaviour from others as is possible. This modelling of ‘normal’ will have an impact. Also, normal expectations, with normal prompts and rewards are in that same environment. This creates a ‘collective efficacy’^23^ which can be defined as “a group's shared belief in its conjoint capabilities to organize and execute the courses of action required to produce given levels of attainment’ (p. 477).^22^ According to Bandura,^23^ there is nothing more effective in influencing people's behaviour than seeing effective practices in use.

A good insight in the practice of family foster care is provided by the excellent documentary that accompanies the Goosens & van de Walle book.^20^ It interviews several *kostgangers* and families and follows them as they go about their daily business. The principles of modelling and positive reinforcement of desirable behaviours and of integration in the community are beautifully demonstrated in this documentary.

## Conclusions

The most positive aspects of the Geel family foster care project are social integration, care in the community and normalisation. The World Health Organization's 2001 report on mental health states: ‘One of the best examples of how communities can become carers of the mentally ill is to be found in the Belgian town of Geel, the site of what is undoubtedly the oldest community mental health programme in the western world.’^24^

In sum, if you had to go into a mental healthcare system, would you rather be going into a family community project like the one in Geel or to an in-patient hospital ward environment?

The family foster care system will not be perfect, but it certainly focuses the mind on what is important in mental healthcare for patients with serious and enduring problems: heart. In the current system and structure in the UK, sometimes the heart is snowed under the tornado of bureaucracy that needs to be completed to demonstrate that one is fully compliant with all the applicable rules and regulations. If one were to apply the ‘modelling’ perspective of the behaviour that nurses model to patients, then it would be completing paperwork in an office.

Mental healthcare is about people helping people – the family foster care project is certainly that. With radical kindness and compassion ordinary people help other ordinary people to lead more fulfilling lives. Belgium is not only the land of chocolates and beer; it is also the birthplace of revolutionary thinking in mental healthcare.

The example of the family foster care project in Geel challenges our view of mental healthcare as a series of interventions and care pathways leading to specific outcomes. Perhaps Geel just offers one intervention: radical compassion and kindness. And the outcomes are lives lived.
